# Elevated Krüppel-like factor 4 transcription factor in canine mammary carcinoma

**DOI:** 10.1186/1746-6148-7-58

**Published:** 2011-10-07

**Authors:** Pei-Yi Chu, Nicholas Chung-Heng Hsu, Albert Taiching Liao, Kun-Tu Yeh, Ming-Feng Hou, Chen-Hsuan Liu

**Affiliations:** 1 Department of Pathology, St. Martin De Porres Hospital, No. 565, Section 2, Daya Road, Chiayi, 60069, Taiwan; 2 Department and Graduate Institute of Veterinary Medicine, School of Veterinary Medicine, National Taiwan University, No. 1, Section 4, Roosevelt Road, Taipei, 10617, Taiwan; 3 Graduate Institute of Medicine, Kaohsiung Medical University, No. 100, Shih-Chuan 1st Road, Kaohsiung, 80708, Taiwan; 4 Department of Pathology, Changhua Christian Hospital, No. 135, Nanxiao Street, Changhua, 500, Taiwan; 5 Cancer Center, Kaohsiung Medical University Hospital, No. 100, Tzyou 1st Road, Kaohsiung, 80708, Taiwan

## Abstract

**Background:**

Krüppel-like factors (KLFs) are critical regulators of biological and physiological systems and have been extensively studied for their roles in cell proliferation, differentiation and survival in the context of cancer. Among the KLFs, KLF4 is highly expressed in human breast cancers and plays an oncogenic role. The present study examined the expression of KLF4 and assessed its significance in canine mammary carcinoma.

**Results:**

Immunohistochemistry was employed to investigate the expression of KLF4 in 142 cases of canine mammary tumor. 75 of the 142 (52.8%) cases were histologically confirmed as mammary carcinoma. Quantification of immunohistochemistry was carried out using Quick score which multiply the staining intensity by the percentage of positive cells. High KLF4 expression was identified in 44 of the 75 (59%) dogs with mammary carcinoma and none in the benign cases. High KLF4 expression occurred only in the tumor cells and not the adjacent normal cells in mammary carcinoma (P < 0.001). Moreover, the high expression level of KLF4 expression was statistically associated with poor grade, late stage, histological subtypes of simple and complex carcinoma, and shorter 24-month survival. The Kaplan-Meier survival analysis also indicated that dogs with high nuclear KLF4 expression had a significantly shorter survival than those with low/moderate KLF4 expression (P = 0.011).

**Conclusions:**

KLF4 is highly and frequently expressed in canine mammary carcinoma and correlates with a more aggressive phenotype.

## Background

Canine mammary tumors are the most common tumor in female dogs. The spontaneous, naturally occurring canine mammary tumors share many features with human breast cancer such as the predominant malignant histological type being adenocarcinoma [1-3] and the expression of estrogen and progesterone receptors (ER/PR), and epidermal growth factor receptor 2 (HER2) in subsets of canine mammary carcinoma [4-7]. It has been suggested that canine mammary carcinomas may be a suitable natural model for the comparative study of human breast cancer [4,5,7-9].

The Krüppel-like factor (KLF) family proteins are transcription factors that play important roles in a wide range of cellular processes, including embryogenesis, proliferation, differentiation, migration, inflammation and tumorigenesis [10-13].

The KLF family consists of 17 different members in which many have been identified as potentially novel oncogenes or tumor suppressors [13,14]. Human KLF4 was first identified using a DNA probe containing the zinc finger region of human erythroid Krüppel-like factor from human umbilical vein endothelial cell cDNA library [15]. The biological effects of KLF4 seem to depend on cancer type rather than unique. KLF4 transcription factor can function as a tumor suppressor and is down-regulated in various human cancer types such as gastric and colorectal cancer [16,17]. On the other hand, high level and oncogenic role of KLF4 were also reported in human breast cancer and oral squamous carcinoma [18,19]. This study investigated the presence of KLF4 and established their clinical significance in canine mammary carcinoma.

## Results

One hundred forty-two dogs (43 Maltese, 11 Yorkshire terriers, 11 Shih-Tzus, 9 Pomeranians, 10 Cocker spaniels, 2 French spaniels, 2 Bichon Frisé, 7 poodles, 2 German shepherd dogs, 1 Shiba, 3 Beagles, 1 Labrador Retriever, 1 Husky, 1 Miniature Doberman, 1 Papillon, 1 Schnauzer, 1 Spitz, and 35 mongrels) were investigated in this study. Of the 142 cases, 52.8% (75/142) were histologically confirmed as carcinoma.

**Figure 1:**
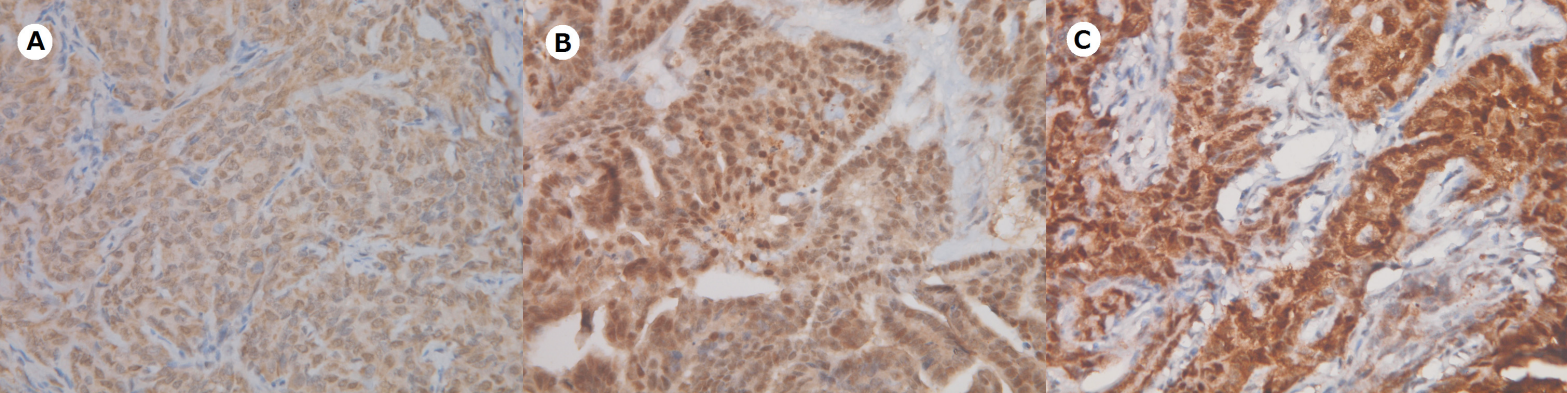
**Representative immunohistochemical staining patterns of KLF4 in canine mammary carcinoma (×400)**. (A) weak (B) moderate (C) strong nuclear KLF4 expression.

**Figure 2:**
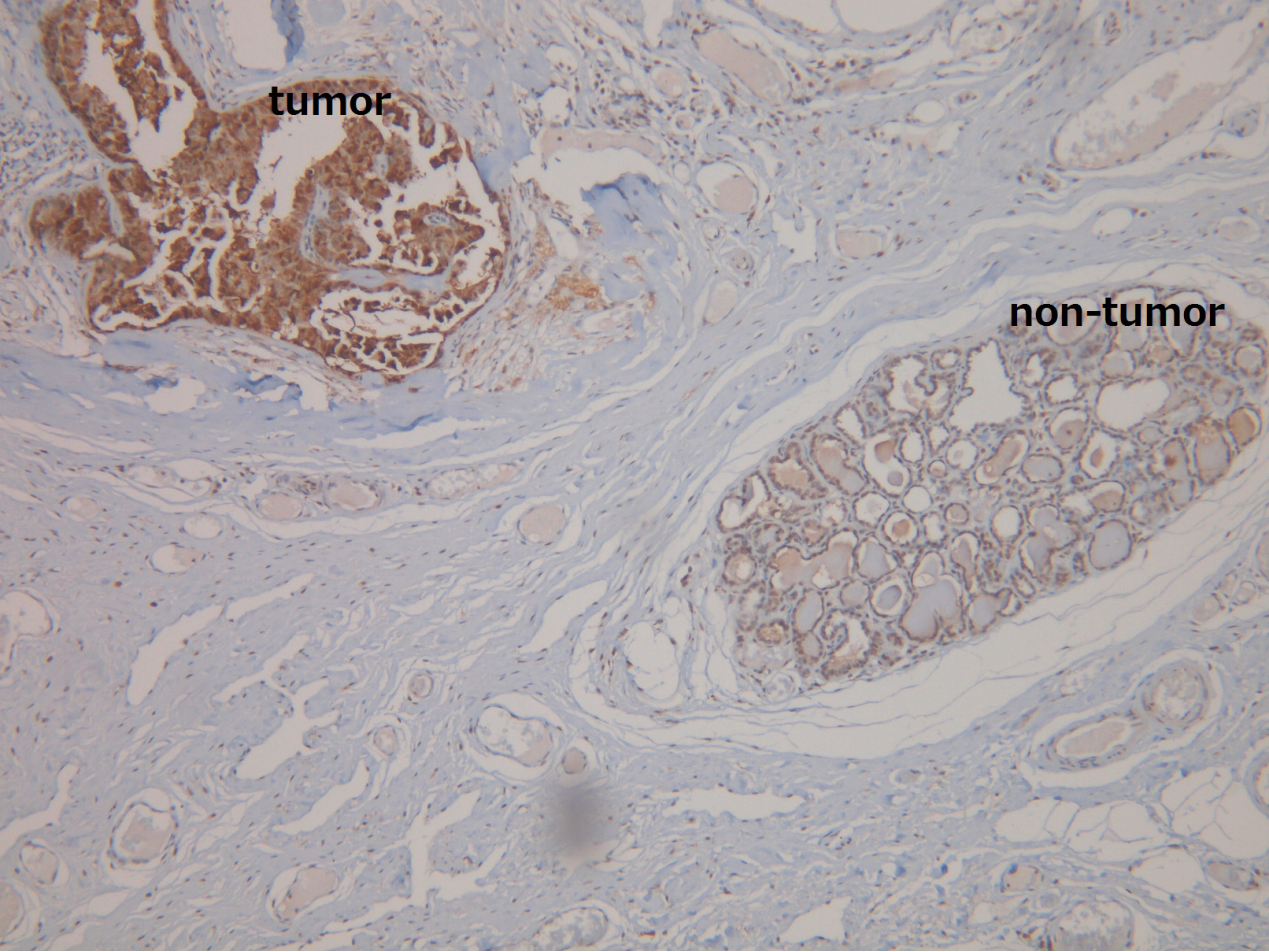
**Elevated KLF4 expression identified in tumor cells and not adjacent non-tumor cells in a representative canine mammary carcinoma (40X)**.

**Figure 3:**
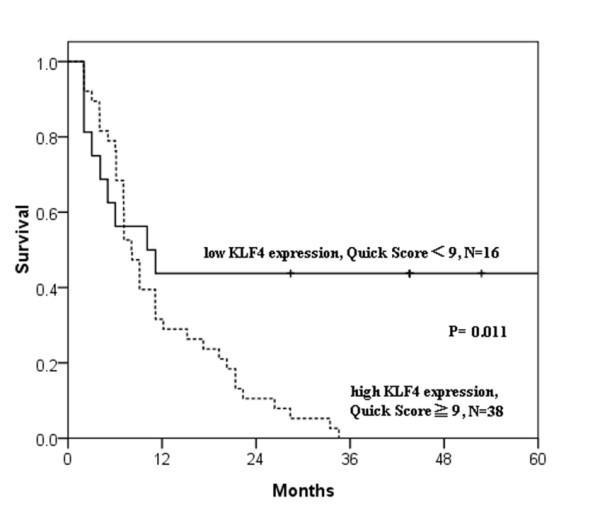
**The Kaplan-Meier plots for survival according to high versus low/moderate nuclear KLF4 expression**. Twenty-one case lacked survival data and were excluded from the analysis.

Analyzing the expression of KLF4 in paraffin-embedded tissues by IHC revealed up-regulated nuclear KLF4 expression in mammary carcinomas as compared to benign tumor cases (Table 1). We divided carcinoma patients into three groups, either high KLF4 expression with Quick score of 9-12, moderate KLF4 expression with Quick score of 5-8, or weak KLF4 expression with Quick score of 1-4 (Figure 1). High expression of KLF4 (as defined by a Quick score of 9 or greater) was identified in 59% (44/75) of dogs with mammary carcinoma and none in the benign tumors. Moreover, high expression level of KLF4 occurred preferentially in the tumor cells and not the adjacent non-tumor cells in mammary carcinoma (P < 0.001, Table 2 and Figure 2). Chi-square analyses for the clinicopathologic characteristics of the 75 canine mammary carcinoma cases in relation to nuclear KLF4 expression showed that high KLF4 expression correlated significantly with shorter 24-month survival (P = 0.01, Table 3). High KLF4 expression was also closely associated with poor grade, late stage, and histological subtypes of simple and complex carcinoma. The Kaplan-Meier survival curves indicated that patients with high nuclear expression of KLF4 had a significantly poor survival than those with low/moderate KLF4 expression as defined by log-rank test (P = 0.011, Figure 3).

**Table 1:** Immunohistochemical quantitation of nuclear KLF4 expression with the Quick score in canine mammary tumor

	Quick score				
		
*Histological classification*	0	1-4	5-8	9-12	total
Benign tumor	0	60	7	0	67
Carcinoma	0	12	19	44	75

**Table 2:** Expression of KLF4 in canine mammary tumor

		Pathological diagnosis			
				
KLF4 expression		Benign tumor	Carcinoma	Total	P
***Tumor part***					
Quick score	< 9	67(100%)	31(41.3%)	98	< 0.001
	≧ 9	0	44(58.7%)	44	
***Non-tumor part***					
Quick score	< 9	67(100%)	75(100%)	142	N/A
	≧ 9	0	0	0	

**Table 3:** Clinicopathologic characteristics of canine mammary carcinoma

	KLF4 expression (Quick score)					
			
	low/moderate (< 9)		High (≧ 9)		N	P
			
	n	%	n	%		
*Age*						
< 12 years	14	45.2%	17	38.6%	31	0.572
≧ 12 years	17	54.8%	27	61.4%	44	
*Ovariohysterectomy*						
No	26	83.9%	33	75.0%	59	0.356
Yes	5	16.1%	11	25.0%	16	
*Tumor Size*						
T1 (< 3 cm)	15	48.4%	11	25.0%	26	
T2 (≧ 3 cm, < 5 cm)	7	22.6%	18	40.9%	25	0.089
T3 (> 5 cm)	9	29.0%	15	34.1%	24	
*Grade*						
I	12	38.7%	5	11.4%	17	
II	14	45.2%	22	50.0%	36	0.009
III	5	16.1%	17	38.6%	22	
*Histological classification*						
Carcinoma in benign tumor	5	16.1%	0	0.0%	5	
Complex carcinoma	14	45.2%	21	47.7%	35	0.020
Simple carcinoma	12	38.7%	23	52.3%	35	
*Location of affected gland*						
cranial	10	32.3%	17	38.6%	27	0.571
caudal	21	67.7%	27	61.4%	48	
*Stage*						
I	15	48.4%	5	11.4%	20	
II	5	16.1%	13	29.5%	18	
III	4	12.9%	7	15.9%	11	0.006
IV	4	12.9%	15	34.1%	19	
V	3	9.7%	4	9.1%	7	
*ER*						
Negative	16	51.6%	23	52.3%	39	0.955
Positive	15	48.4%	21	47.7%	36	
*PR*						
Negative	6	19.4%	5	11.4%	11	0.509
Positive	25	80.6%	39	88.6%	64	
*Her-2/neu*						
Negative	26	83.9%	32	72.7%	58	0.281
Positive	5	16.1%	12	27.3%	17	
*Molecular phenotyping*						
Basal	10	32.3%	15	34.1%	25	
HER-2 overexpressing	2	6.5%	5	11.4%	7	
Luminal A	12	38.7%	14	31.8%	26	0.761
Luminal B	3	9.7%	7	15.9%	10	
Null	4	12.9%	3	6.8%	7	
*Survival^a^*						
< 24 months	9	56.3%	34	89.5%	43	0.010
≧ 24 months	7	43.8%	4	10.5%	11	

^a ^Twenty-one cases lacked survival data and were excluded from the analysis.

## Discussion

Studies of KLF proteins in mouse models of human diseases have revealed the normal biological roles of the KLFs as well as their involvement in the pathogenesis of a variety of diseases such as cancer [20]. Previous studies have shown that approximately 70% of human breast cancer has increased KLF4 expression and that up-regulated nuclear KLF4 expression is associated with a more aggressive phenotype [18,21]. The oncogenic properties of KLF4 in breast cancers was also confirmed in vitro and using xenograft tumor model in which KLF4 knockdown inhibited breast cancer development [22].

The breast cancer stem cell hypotheses suggest that breast cancer is derived from a single cell with stem-like properties that is capable of tumor initiation and formation. KLF4 can inhibit differentiation and increase self-renewal in embryonic stem (ES) cells [23,24]. Forced expression of KLF4, along with transcription factors, Oct4, c-myc, and Sox2, can reprogram or dedifferentiate somatic cells into

induced pluripotent stem cells (iPSCs) in both mice [25,26] and human [27-29]. Taken together, these finding suggest that KLF4 is indispensable for the regulation of stem cells and contributes to tumorigenesis.

In this study, we investigated the expression and clinical relevance of KLF4 in canine mammary carcinoma. Immunihistochemistry revealed that nuclear expression of KLF4 was elevated in tumor cells of canine mammary carcinoma. Although increased KLF4 expression was not related to prognostic markers such as ER, PR or HER2. High nuclear KLF4 expression was associated significantly with a more aggressive phenotype as indicated by poor grade, late stage, histological subtypes of simple and complex carcinoma, and shorter 24-month survival in canine mammary carcinoma. Despite diffuse cytoplasmic KLF4 expression with different degree of intensity was observed among the samples. The cytoplasmic KLF4 expression was not related to any clinicopathologic parameters and survival (data not shown). The Kaplan-Meier survival analysis also indicated that dogs with high nuclear expression of KLF4 had a significantly shorter survival as compared with ones with low/moderate nuclear KLF4 expression.

We provided evidence for the first time that KLF4 is preferentially and highly expressed in canine mammary carcinoma. As in human breast cancer, KLF4 plays an oncogenic role in canine mammary carcinoma. Further studies are needed to validate whether systemic targeting of KLF4 would inhibits the oncogenic functions of KLF4 thus provides an effective strategy for the treatment of canine mammary carcinoma.

## Conclusions

Nuclear expression of KLF4 is frequently elevated in canine mammary carcinoma and closely correlated with a more aggressive phenotype and shorter survival.

## Methods

### KLF4 Immunohistochemistry

Paraffin-embedded tissue blocks of 142 cases of canine mammary tumor diagnosed between January 2003 and April 2008 were retrieved from the archives of the School of Veterinary Medicine, National Taiwan University, Taiwan. The tumors were diagnosed according to the WHO criteria for canine mammary neoplasms [30]. Samples were first de-waxed in xylene and re-hydrated through graded alcohols, followed by a rinse using 10 mM Tris-HCl (pH 7.4) and 150 mM sodium chloride, then treated with 3% hydrogen peroxide for 5 min. Slides were incubated with 1:250 dilution of anti-KLF4 antibody (sc-20691, Santa Cruz Biotechnology, USA) for 1 hour at room temperature, then thoroughly washed three times with PBS. Bound antibodies were detected using the LSAB+ kit (Dako, USA). The slides were then counterstained with haematoxylin stain solution. Paraffin-embedded sections of human breast cancer cells of homogeneous KLF4 immunophenotype were included as positive controls. Negative controls had the primary antibody omitted and replaced by PBS. Quantification of KLF4 expression was carried out using Quick score which multiply the staining intensity by the percentage of positive cells [31-33]. The intensity of staining was scored as 0, 1, 2, and 3 standing for negative, weak, moderate, and strong staining, respectively. The percentage of tumor cells staining positively was scored as follows: 0 = 0%, 1 = 1-25%, 2 = 26-50%, 3 = 51-75%, and 4 = 76-100%, compared with the total of tumor cells. The immunohistochemical results were evaluated by two investigators scoring independently. Conflicting scores were resolved at a dual head microscope.

### Molecular Phenotyping

Immunohistochemistry was performed in parallel as described above with monoclonal antibodies for ER (1:35 dilution, Dako, Denmark), PR (1:200 dilution, Thermo Scientific, USA), HER2 (1:400 dilution, Dako, Denmark), CK5 (1:100 dilution, Novacastra, UK), and P-cadherin (1:100 dilution, Novacastra, UK). ER and PR immunoreactivity was considered positive when more than 10% of the neoplastic cells expressed this marker [5]. HercepTest scoring system was applied to evaluate HER2 expression (0 = no staining or membrane staining in fewer than 10% of tumor cells; 1+ = faint, barely perceptible membrane staining in more than 10% of tumor cells; 2+ = weak to moderate complete membrane staining observed in more than 10% of tumor cells; 3+ = strong and complete membrane staining in more than 10% tumor cells) [5]. In this study, overexpression of HER2 was defined as a HercepTest score of 3+. As for CK5 and P-cadherin, cytoplasmic staining in > 50% of cells was considered positive [5]. Immunohistochemical panel which involved the evaluation of ER, HER2, CK5, and P-cadherin was used to distinguish canine mammary carcinoma subtypes [5,34].

### Statistical Analysis

Overexpression of KLF4 was defined as a Quick score of 9 or greater on the scale of 0 to 12. Patterns and correlations of KLF4 and clinicopathologic parameters of canine mammary tumor were examined by Pearson's chi-square test. Survival rate was calculated using Kaplan-Meier analysis and compared by the Cochran-Mantel-Haenszel test (log-rank test). Survival was defined as the time between date of diagnosis and date of death. Subjects still alive at the end of the study were censored at the date of last follow-up. Cases that lacked survival information were excluded from the analysis. A P value of less than 0.05 was considered to indicate statistical significance.

## Authors' contributions

PYC drafted the manuscript, NCH performed the statistical analysis, ATL and KTY carried out the immunohistochemical staining, MFH and CHL designed the study. All authors read and approved the final manuscript.
